# Contact-induced apical asymmetry drives the thigmotropic responses of *Candida albicans* hyphae

**DOI:** 10.1111/cmi.12369

**Published:** 2014-11-25

**Authors:** Darren D Thomson, Silvia Wehmeier, FitzRoy J Byfield, Paul A Janmey, David Caballero-Lima, Alison Crossley, Alexandra C Brand

**Affiliations:** 1 School of Medical Sciences, University of AberdeenAberdeen, AB25 2ZD, UK; 2 Institute for Biology Valrose, University of Nice-Sophia AntipolisNice, France; 3 Institute for Medicine and Engineering, University of PennsylvaniaPhiladelphia, PA, USA; 4 Department of Molecular Biology and Biotechnology, Sheffield UniversitySheffield, S10 2TN, UK; 5 Department of Materials, Oxford UniversityOxford, OX1 3PH, UK

## Abstract

Filamentous hyphae of the human pathogen, *Candida albicans*, invade mucosal layers and medical silicones. *In vitro*, hyphal tips reorient thigmotropically on contact with small obstacles. It is not known how surface topography is sensed but hyphae lacking the cortical marker, Rsr1/Bud1, are unresponsive. We show that, on surfaces, the morphology of hyphal tips and the position of internal polarity protein complexes are asymmetrically skewed towards the substratum and biased towards the softer of two surfaces. In nano-fabricated chambers, the Spitzenkörper (Spk) responded to touch by translocating across the apex towards the point of contact, where its stable maintenance correlated with contour-following growth. In the *rsr1*Δ mutant, the position of the Spk meandered and these responses were attenuated. Perpendicular collision caused lateral Spk oscillation within the tip until after establishment of a new growth axis, suggesting Spk position does not predict the direction of growth in *C. albicans*. Acute tip reorientation occurred only in cells where forward growth was countered by hyphal friction sufficient to generate a tip force of ∼ 8.7 μN (1.2 MPa), more than that required to penetrate host cell membranes. These findings suggest mechanisms through which the organization of hyphal tip growth in *C. albicans* facilitates the probing, penetration and invasion of host tissue.

## Introduction

Pathogenic fungi have developed growth and exploration strategies relevant to their host environment. Once adhered to the host, most fungi produce invasive filaments which, like *Uromyces appendiculatus*, navigate host surfaces by mechanosensing in order to identify suitable entry points. Some respond to the specific topography of leaf stomata, on which they form a specialized structure called an appressorium. Through strong adhesion to the host leaf, the enormous internal turgor pressure generated by appressoria is transmitted as force against the host leaf in the form of a penetration peg (Allen *et al*., 1991; Howard *et al*., 1991; Hoch *et al*., 1993). These contact-dependent (thigmotropic) growth behaviours can be induced by growing fungi on inert substrates that mimic host topography. *In vitro* systems can therefore be useful in identifying growth behaviours that are relevant to the host infection process. The identification of fungal growth traits that facilitate invasion in humans is hampered by the highly heterogeneous nature of the various body sites from which fungi can be isolated. *Candida albicans* normally lives as a commensal yeast on mucosal surfaces, but suboptimal containment of the fungus by the host immune system induces morphogenesis and the production of hyphae (Odds, 1988). Hyphal filaments invade and disrupt the outer epithelial cell layers (e.g. in oral or vaginal ‘thrush’) or facilitate escape from the vasculature into solid organ tissue during hospital-acquired bloodstream infections. They do this by active penetration of host tissue or by germinating within host cells that have internalized the yeast form of the fungus (Dalle *et al*., 2010; Wächtler *et al*., 2011). Both invasion mechanisms involve breaching of the host cell membrane, but the importance of physical force in this process is not known. However, the ability of hyphae to regulate their direction of growth is important for tissue penetration because deletion of Rsr1, a cortical landmark protein that positions the activity of cell polarity complexes, attenuates virulence and cell damage (Yaar *et al*., 1997; Brand *et al*., 2008).

The polarity complexes are well conserved in filamentous fungal cells and consist of the Spitzenkörper (Spk), the polarisome and the exocyst. The Spk, or ‘apical body’, was first observed by light microscopy as a dynamic dark zone just behind the hyphal tip (Girbardt, 1957). Electron and fluorescence microscopy using the lipid stain, FM4-64, showed it to comprise a compact cloud of secretory vesicles (Grove and Bracker, 1970; Howard, 1981; Fischer-Parton *et al*., 2000) and in *C. albicans* it has been visualized through the localization of fluorescently tagged Myosin light chain 1 (Mlc1) and Bni1, a motor protein and an actin nucleator, respectively (Crampin *et al*., 2005; Li *et al*., 2005). The Spk functions in conjunction with the polarisome, a collection of proteins that appears as a crescent at the apical plasma membrane (Crampin *et al*., 2005; Jones and Sudbery, 2010). The polarisome contains the Spa2 cell-end anchoring protein, Cdc42, a small GTPase that activates actin-mediated polarized vesicle delivery, Exo70, a membrane-associated component of the exocyst that determines secretory vesicle docking sites, and Kel1, a putative cell-end marker homologous to TeaA in filamentous fungi (Fujiwara *et al*., 1998; Zheng *et al*., 2003; Takeshita *et al*., 2008; Jones and Sudbery, 2010; Gutiérrez-Escribano *et al*., 2011). In models of hyphal growth, the Spk has been termed the Vesicle Supply Centre and placed symmetrically within the hyphal apex, from whence vesicle diffusion determines the hyphoid curve of the cell apex (Bartnicki-Garcia *et al*., 1989; Gierz and Bartnicki-Garcia, 2001; Li *et al*., 2005). This positioning is likely true for cells growing within a homogenous environment, such as in liquid media or during embedded growth in agar, but an asymmetrical, or ‘nose-down’, tip morphology has been observed in several fungi during growth on surfaces (Read *et al*., 1992; Bowen *et al*., 2007). The positioning of the Spk close to the substratum has also been recorded (Kwon and Hoch, 1991; Dijksterhuis, 2003) and a role for the Spk in touch responses was proposed when it was observed to move away from the point of contact in the rust fungus, *U. vignae* (Dijksterhuis, 2003). Spk regulation and behaviour may therefore be closely linked to directional growth responses and it has been proposed that the position of the Spk predicts the site of new tip growth (Bracker *et al*., 1997; Riquelme *et al*., 1998).

Previous *in vitro* studies, in which hyphae were free to grow in the X, Y and Z dimensions, showed that *C. albicans* hyphae exhibit thigmotropic tip reorientation in response to small topographical ridges less than half the height of a hypha (Watts *et al*., 1998; Brand *et al*., 2007). How do fungal cells, which are surrounded by a stiff cell wall, sense such small features? Deletion of the Rsr1 polarity-site marker abolished all tip reorientation responses (Brand *et al*., 2008), but its role in this process has not been further elucidated. Here we used live cell imaging and nano-fabricated chambers to observe how hyphal tips interact with obstacles in space and time and conclude that the cellular organization of hyphal growth in *C. albicans* imparts specific characteristics important for the exploration, penetration and invasion of its host environment.

## Results

### *C. albicans* hyphae grow with an asymmetrical morphology and a bias towards softer surfaces

**Figure 1:**
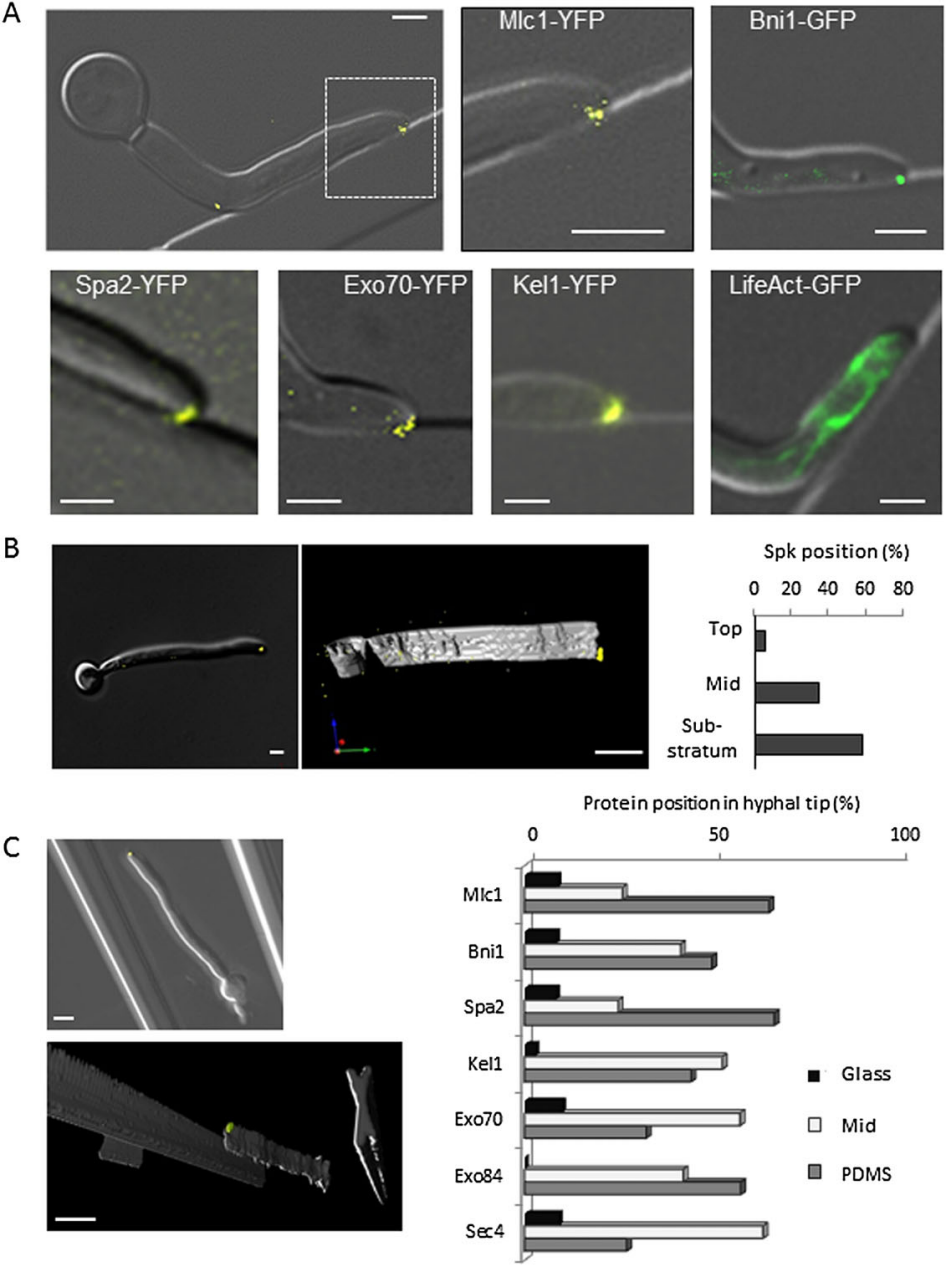
Hyphal tip contact induces asymmetric morphology and polarity-complex positioning.A. Hyphae were grown in live cell imaging chambers featuring straight and curved barriers. Merged Differential Interference Contrast (DIC) and fluorescence images of polarity complex markers: Mlc1-YFP and Bni1-GFP (a formin) mark the Spk, Spa2-YFP and Kel1-YFP mark the polarisome and Exo70-YFP marks the exocyst. LifeAct indicates actin cables, absent in the extreme apex.B. Hyphae expressing Mlc1-YFP were grown on glass or PDMS surfaces in Petri dishes and Z-stack images generated in DIC and fluorescence. Images were 3D (three-dimensionally)-rendered using Volocity software and the position of the Spk relative to the substratum was determined (top: away from the substratum, mid: central position, substratum: adjacent to the substratum, *P* = < 0.03, *n* = 18).C. Hyphae expressing fluorescently-tagged polarity complex markers were grown between glass and PDMS surfaces in live cell imaging chambers. Hyphae were imaged as for (B). The position of the complexes relative to the substrata was determined (*P* = < 0.001, *n* = 209). Bars = 2 μm.

To visualize hyphal tip morphology and growth behaviour in real time, we grew cells in nano-fabricated live cell imaging chambers featuring curved and angled shapes, gaps and channels with which to test fungal responses (Fig. S1). In this system, changes in hyphal growth direction were confined to the two-dimensional horizontal plane. When extending hyphal tips made contact with an obstacle, hyphae reoriented their growth axis along the contour of the barrier, which permitted direct observation of the hypha-substrate interface in the horizontal plane (Fig. 1A; Movie S1). DIC microscopy showed that *C. albicans* hyphal tips adopted an asymmetric morphology towards the barrier after reorienting. Strains carrying fluorescently-tagged proteins were used to visualize the relative positioning of the key intracellular polarity complexes within the skewed tip. Mlc1-YFP and the actin–cable-nucleating formin, Bni1-GFP, were used as markers for the Spk. Spa2-YFP represented the polarisome, and the exocyst was represented by Exo70-YFP (Crampin *et al*., 2005; Li *et al*., 2005; Takeshita *et al*., 2008). Kel1, a homolog of the *Aspergillus nidulans* microtubule-associated cell-end marker, TeaA, was also visualized in addition to actin cables, which associate with the LifeAct-GFP construct (Gutiérrez-Escribano *et al*., 2011; Sudbery, 2011). The intracellular positioning of the polarity complexes, while retaining their longitudinal positions relative to each other, was asymmetrically skewed towards the barrier, consistent with the overall tip morphology (Fig. 1A). Actin cables were visible sub-apically but not within the hyphal tip. To establish whether the position of polarity proteins was asymmetrically skewed in the vertical (Z) plane during growth on a surface, hyphae were germinated on glass or silicone and incubated in a Petri dish instead of within the confines of live cell imaging chambers. Z-stack microscopy and three-dimensional image-rendering software were used to score the position of the Spk as being ‘top’, ‘mid’ or ‘substratum’ (i.e. adjacent to the solid base) (Fig. 1B). On both surfaces, the Spk was oriented towards the substratum (59%, *P*  <  0.03). The ‘nose-down’ tip shape and Spk position are therefore general features of *C. albicans* hyphal growth on surfaces.

To examine the role of physical properties of the substratum in the skewing of polarity proteins, hyphae carrying fluorescently tagged protein complexes were grown between silanized glass and silicone elastomer, with the silicone uppermost on an inverted microscope (Fig. 1C). Both materials had similarly hydrophobic surface chemistries, with a water contact angle of 92 ± 5° and 106 ± 3°, respectively, but glass is a much stiffer material, with a Young's modulus several orders of magnitude higher than silicone (Armani *et al*., 1999; Seal *et al*., 2001). The polarity complex proteins were centrally localized (48%) or skewed towards the silicone (45%), while only 7% oriented towards the glass, suggesting that hyphal tips are sensitive to the mechanical properties of the substratum.

### Asymmetrical hyphal tips follow contours and grow into gaps

**Figure 2:**
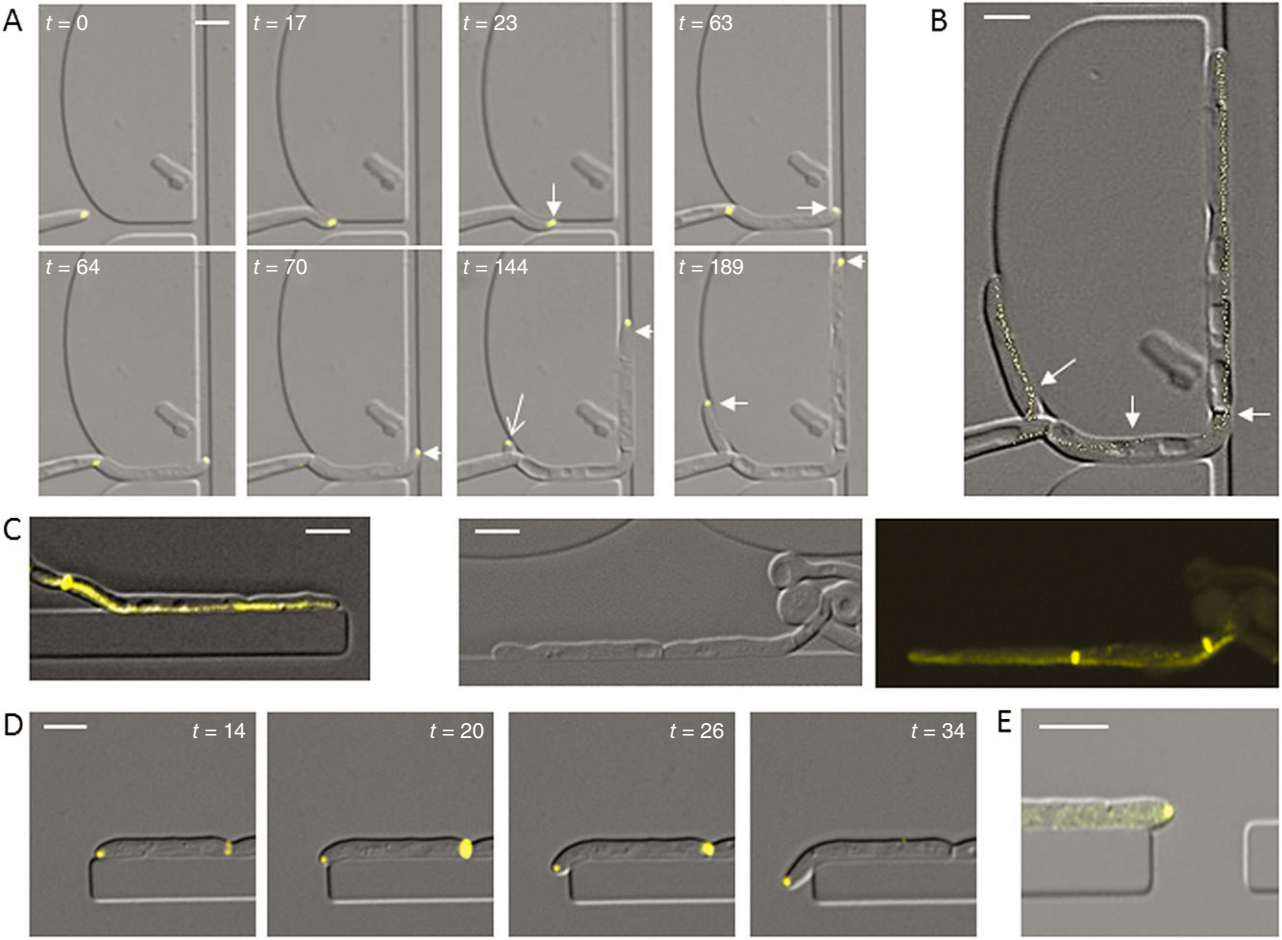
Contact-induced tip asymmetry promotes growth along contours.A. The Spk translocates across the hyphal apex in response to new tip contact (Movie S2). Hyphae were grown in a live cell imaging chambers and imaged using DIC and fluorescence microscopy. The Spk became asymmetrically localized after tip contact with an external barrier (arrows).B. Plot of Mlc1-YFP fluorescence signal maxima at 1 min intervals superimposed on the end point of the movie shown in (A) indicates stable positioning of the Spk adjacent to the external contour. Arrows indicate Spk translocation.C, D. Tracking of Mlc1-YFP during growth along a barrier showed stable positioning of the Spk, including during presumptum formation. Tip asymmetry promotes contour following around gentle (A) and sharp (D) curves.E. Hypha with a symmetrical tip does not follow barrier contour. Bars = 5 μm.

To examine the dynamics of Spk responses, imaging chambers were used to track the position of Mlc1-YFP during hyphal growth through channels that induced sequential tip reorientation. On encountering an obstacle, the Spk translocated towards the site of contact (Fig. 2A; Movie S2). On encountering a second obstacle, the Spk translocated across the hyphal apex towards the new point of contact. A plot of Spk position at 1 min intervals (Fig. 2B) showed that the Spk consistently maintained its position during growth along a contour and was not perturbed by the formation of a presumptum, a nascent septin ring that is laid down at the cell periphery to later become a sealed septum after nuclear division across the site (Fig. 2C) (Finley and Berman, 2005). Hyphal growth followed curved contours, and even sharp bends, in a direction consistent with Spk bias (Fig. 2A and D). In contrast, hyphae that germinated adjacent to a barrier grew with symmetrical tip morphology in the X-Y plane, had a central Spk and maintained a straight growth trajectory (Fig. 2E). Spk positioning was therefore dependent on direct tip contact and its asymmetry correlated with the direction of growth in response to topographical features.

### Deletion of Rsr1 attenuates contact-dependent hyphal behaviours

**Figure 3:**
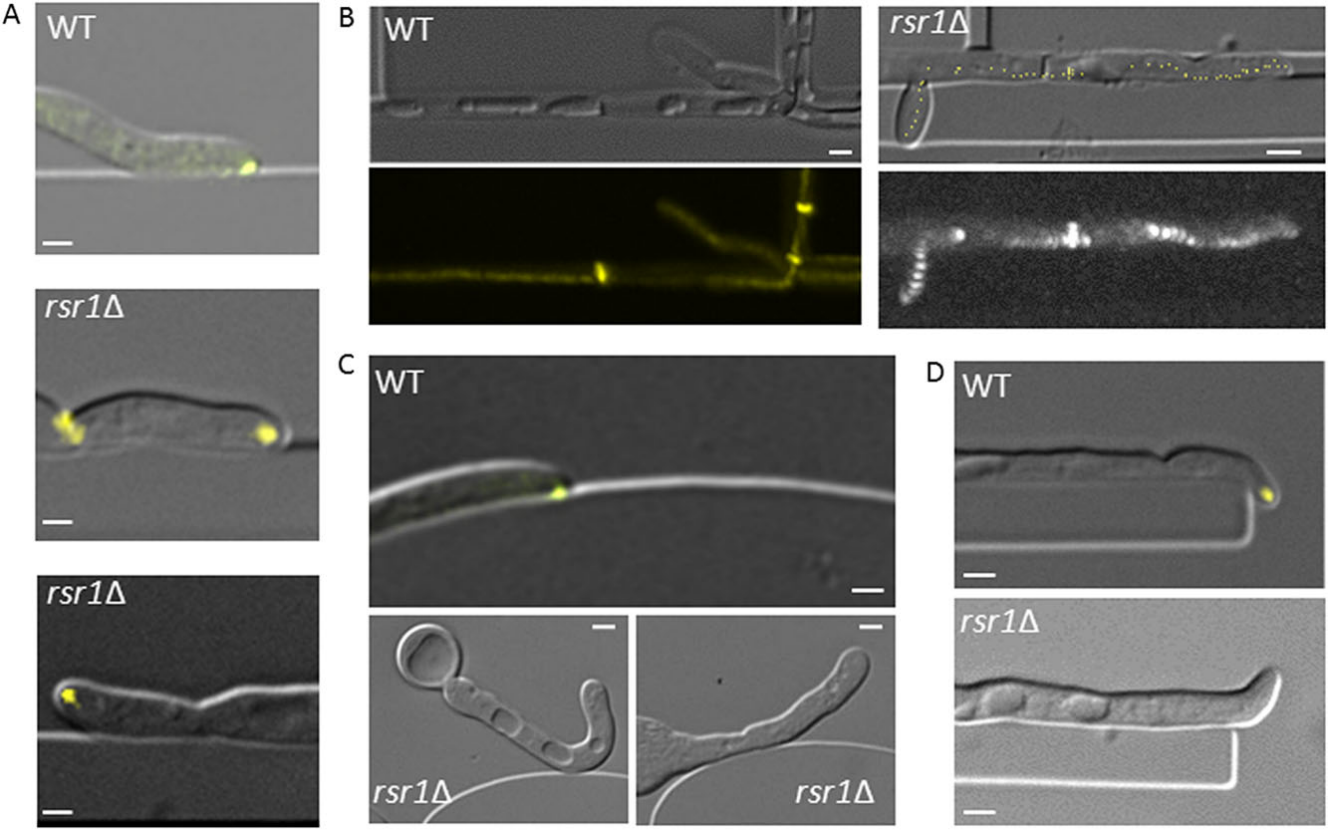
Deletion of Rsr1 alters tip morphology, stable Spk positioning and hyphal contour following. A. Tip morphology and Spk position (Mlc1-YFP) responses in hyphae of the wild-type and *rsr1*Δ null mutant grown in live cell imaging chambers.B. Plots of Mlc1-YFP show time lapse tracking of the Spk in wild-type and *rsr1*Δ mutant cells during hyphal growth along contours.C, D. Contour following in wild-type and *rsr1*Δ mutant hyphae, grown and imaged as above. Bars = 2 μm.

Rsr1 is a regulator of directional growth in yeast, and its deletion in *C. albicans* causes erratic movement of the Spk in hyphae (Yaar *et al*., 1997; Hausauer *et al*., 2005). To explore the role of Rsr1 in contact-dependent growth responses, the *rsr1*Δ strain carrying Mlc1-YFP was imaged during hyphal growth in live cell imaging chambers. In contrast to wild-type cells, after contact-dependent tip reorientation *rsr1*Δ hyphal tips did not sustain an asymmetrical morphology and became rounded (Fig. 3A). Time lapse plots of Spk localization showed greater variation in Spk trajectory compared with wild-type cells (Fig. 3B). *rsr1*Δ hyphae did not consistently follow topographical contours and instead grew away from barriers in a random manner, which was never seen in wild-type cells (Fig. 3C). The hyphae of the *rsr1*Δ mutant were therefore less likely to grow around obstacles and into gaps (Fig. 3D).

**Figure 4:**
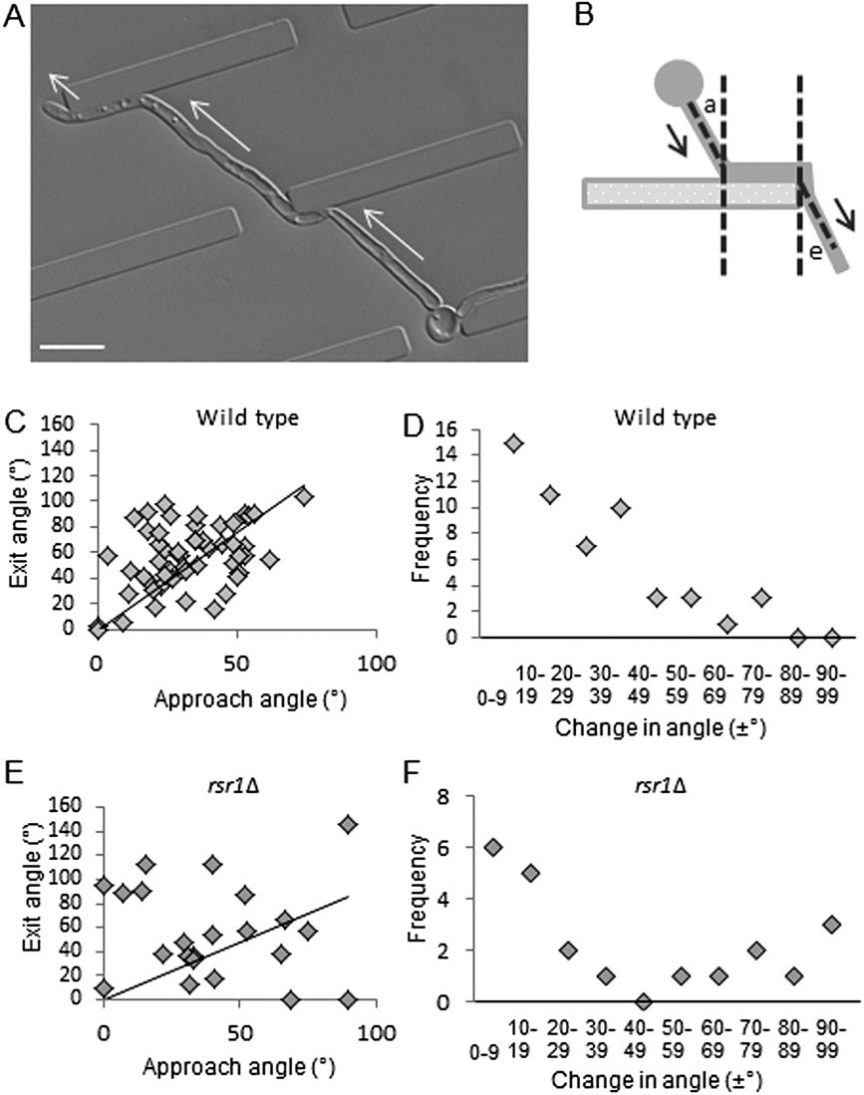
Wild-type hyphae, but not the *rsr1*Δ mutant, maintain an overall growth trajectory.A. Hyphae were grown in live-cell imaging chambers featuring barriers with gaps. Hyphae resumed the original growth trajectory (arrows) after temporary deflection by an obstacle (Movie S3). Bar = 10 μm.B. Scheme for measurement of hyphal angle of approach (a) and angle of exit (e) relative to a barrier.C, E. Scatter plot of angle of approach versus angle of exit for wild-type (*P* = 0.007, *n* = 52) and *rsr1*Δ hyphae (*P* = 0.57, *n* = 22).D, F. Frequency plots of trajectory changes of increasing magnitude for wild-type and *rsr1*Δ hyphae.

After circumnavigating an obstacle, wild-type hyphae reverted to their original growth trajectory as soon as possible (Fig. 4A; Movie S3). This response was quantified by measuring the change between the angle of approach (towards the obstacle) and the angle of exit (away from the obstacle) in individual hyphae (Fig. 4B and C). In wild-type cells, large net alterations in the overall growth trajectory were infrequent and most hyphae altered course by less than 20° (Fig. 4D; *P* = 0.007). In contrast, hyphae of the *rsr1*Δ mutant were attenuated in their ability to maintain an overall growth trajectory and instead made pronounced and random directional changes (Fig. 4E and F). Taken together, these results suggest that deletion of Rsr1 uncouples polarity positioning determinants from external directional growth cues.

### Perpendicular contact causes Spk oscillation and generates tip force

**Figure 5:**
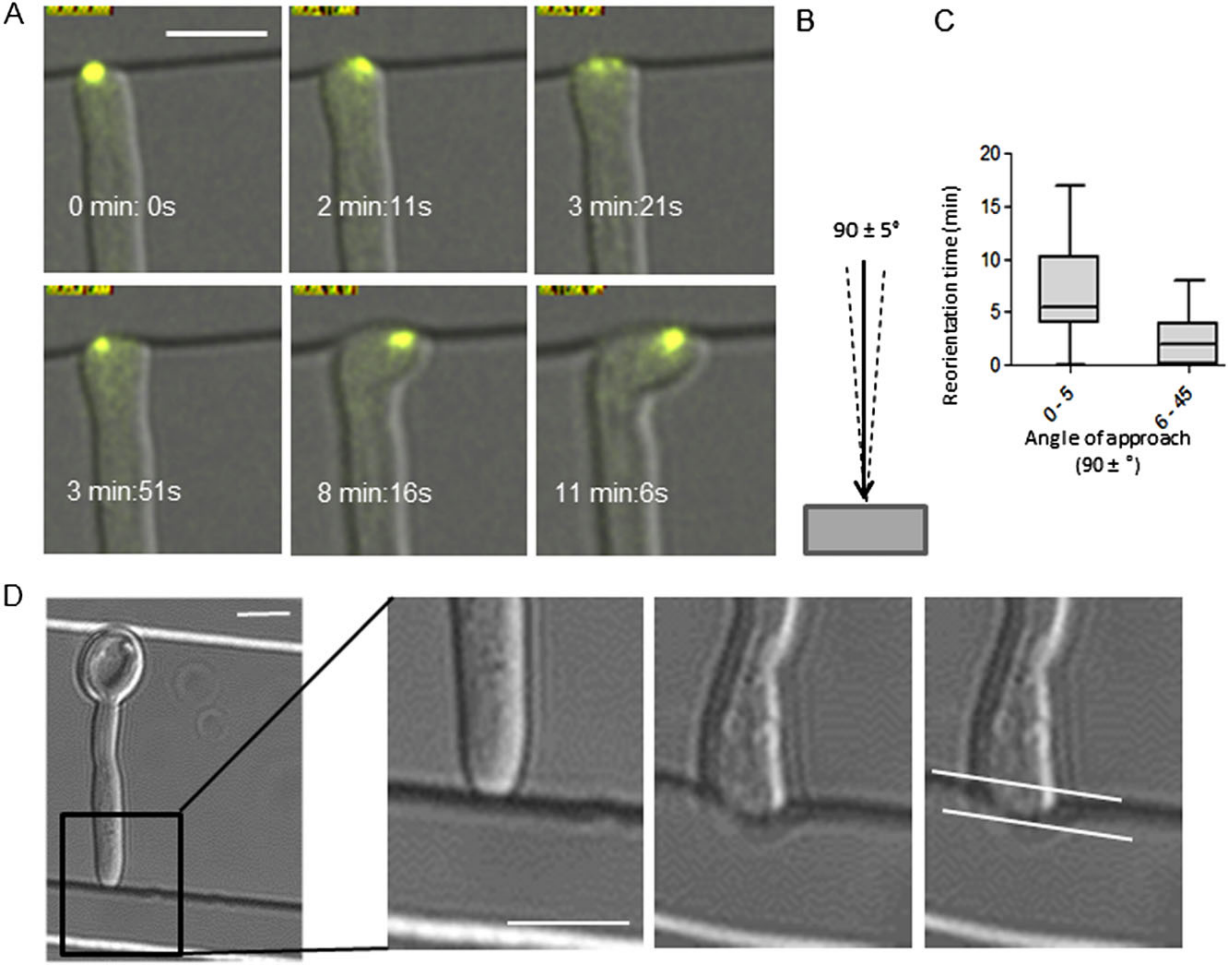
Perpendicular collision of the hyphal tip with an obstacle induces Spk oscillation and generates hyphal tip force.A. Time lapse images of Spk oscillation in hyphal tips that contacted a barrier perpendicularly (B, defined as an angle of 90 ± < 5°) (Movie S4).C. Duration of reorientation of hyphal tips undergoing perpendicular contact compared with all other angles of approach (*P* ≤ 0.0001, *n* = 74). D. Hyphal tips generated a mean indentation distance in PDMS of 0.95 ± 0.04 μm (*n* = 61). Bars = 5 μm.

On tangential hyphal tip contact, the Spk smoothly translocated across the hyphal apex but perpendicular tip collisions (i.e. with an angle of approach of 90  ± < 5°) caused lateral oscillations of the Spk and delayed hyphal reorientation (Fig. 5A–C; Movie S4). The emergence of new tip growth along the barrier occurred while the Spk was still oscillating, suggesting that Spk position is not an absolute predictor of the direction of growth (see below). Prior to reorientation, the force exerted by the hyphal tip caused indentation of the polydimethylsiloxane (PDMS) elastomer, permitting the force exerted by its hyphal tips to be quantified for the first time. A standard curve of force versus indentation distance was generated using atomic force microscopy to plot the force required to indent PDMS to increasing depths using a cantilevered 2 μm diameter glass bead to mimic the hyphal tip (Fig. 5D; Fig. S2). The depths of indentations made by wild-type hyphae in PDMS were measured and the applied force was extrapolated from the standard curve. The mean indentation distance for wild-type hyphae was 950 ± 40 nm, which generated a tip force of ∼ 8.7 μN and a pressure of 1.2 MPa. The force applied by the *rsr1*Δ strain was not significantly different at 8 μN, although its increased hyphal diameter (2.7 vs. 2.4 μm, *P* = 0.001) slightly reduced the pressure applied to 1.1 MPa. The attenuated virulence seen in the *rsr1*Δ strain is therefore unlikely to be due to its inability to apply pressure to host tissue.

### Frictional force is required between hypha and substrate for acute tip reorientation

**Figure 6:**
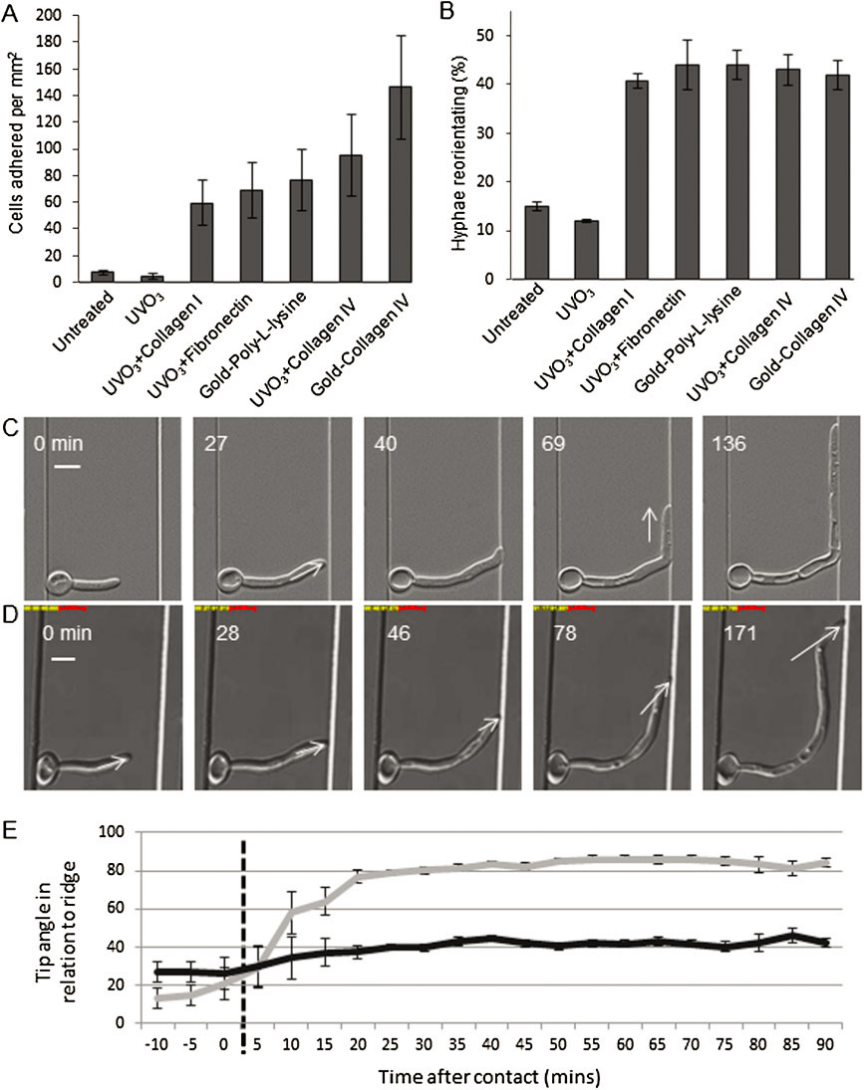
Frictional force between hypha and substratum is required for acute tip reorientation.A. Adhesion of wild-type *C. albicans* cells on PDMS samples with differentially modified surface chemistries. Modified PDMS samples were inoculated with standardized cell concentrations and the number of cells adhered per mm^2^ per PDMS surface was determined. Bars = standard error of the mean (SEM), *n* = 3.B. Hyphal reorientation assays were carried out by adhering wild-type cells to the same series of PDMS surface chemistries and incubating in Petri dishes, where hyphae were free to grow in X, Y and Z planes. The response to ridges 1 μm high was expressed as the percentage of hypha-ridge interactions that resulted in hyphal reorientation. Bars = SEM, *n* = 3.C. Time lapse of immobilized hypha reorienting on contact with a barrier in an enclosed imaging chamber 1 μm in height (Movie S1). Bar = 5 μm.D. Time lapse of a non-immobilized hypha contacting a barrier in an enclosed chamber 1.5 μm in height (Movie S5). Bar = 5 μm.E. Plot of the hyphal tip angles relative to the barrier for the immobilized hypha in panel C (arrows) and the non-immobilized hypha in panel D (arrows). The time of contact with the barrier is designated as *t* = 0 min. Error bars = SEM, *n* = 17.

Previous experiments had indicated that poorly adhered cells do not exhibit a normal level of thigmotropic response. To examine the role of cell adhesion in *C. albicans* hyphal tip reorientation, the thigmotropic responses of wild-type hyphae were quantified by growing cells on PDMS samples that had been chemically modified to generate seven surface chemistries with increasing capacity to bind *C. albicans* cells (Fig. 6A). The surfaces also featured ridges of 1 μm in height to elicit thigmotropic responses (Brand *et al*., 2008). Cell adhesion was poorest on untreated and UV-ozone (UVO_3_)-treated PDMS but increased on surfaces coated with extracellular matrix-associated proteins. The strongest adhesion was observed for Collagen IV-coated surfaces, consistent with previous studies (Yan *et al*., 1998). Adhesion rates were 30-fold higher on PDMS sputtered with gold and coated with Collagen IV than on untreated surfaces. In thigmotropism assays, yeast cells were adhered to these surfaces and hyphae grown by incubation in liquid medium in Petri dishes. In this system, hyphae were free to grow in three dimensions (X, Y and Z), so could grow over the ridges. Surfaces with low adhesion rates induced little hyphal reorientation. A 10-fold increase in adhesion induced a threefold increase in hyphal reorientation (Fig. 6B) and this remained constant at higher adhesion rates, indicating that a threshold level of cell adhesion was required to generate hyphal reorientation. To explore this further, hyphae were imaged in 1 μm live cell imaging chambers where cells were immobilized through the frictional pressure of the coverslip rather than by cell adhesion. Although immobilized hyphae reoriented by 90° on perpendicular contact with a barrier (Fig. 6C; Movie S1), cells in deeper chambers were exposed to less frictional force and underwent sub-apical bending in preference to tip reorientation. In these conditions, the angle of approach was maintained by the tip in relation to the obstacle (Fig. 6D; Movie S5). This demonstrates that tip contact alone is insufficient to trigger hyphal reorientation and, instead, reorientation in *C. albicans* requires a degree of mechanical stress (Fig. 6E). This is generated by frictional or adhesive force along the length of the hypha that is sufficient to counter the backward thrust of forward tip growth and prevent dissociation of the cell from the substratum.

### Spk position correlates with specific directional responses but is not a predictor of growth direction

**Figure 7:**
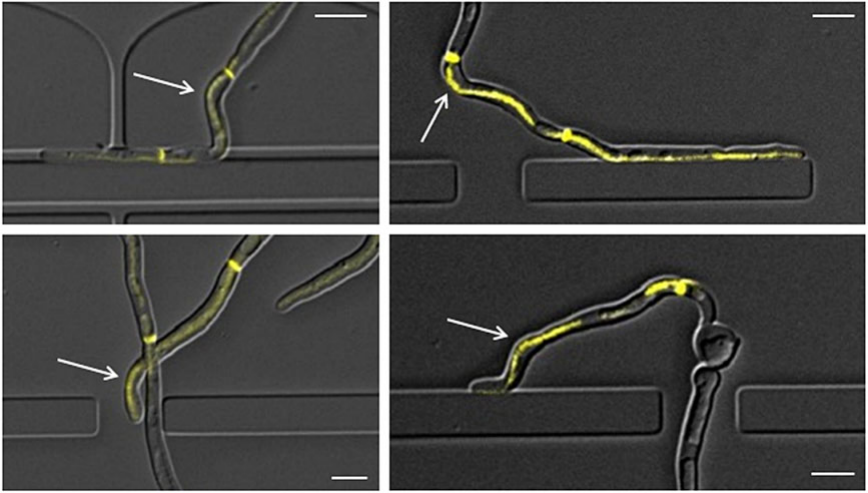
The Spk is not the absolute determinant of growth direction. Hyphae expressing the fluorescently-tagged Spk marker protein, Mlc1-YFP, were grown in live cell imaging chambers. The sequential positions of the Spk were mapped over time and the plot overlaid onto the end point DIC image of the hypha. Examples of sites where the SPK is positioned on the opposite side of the hypha in relation to the growth direction are indicated (arrows). Bars = 5 μm.

In this study, the asymmetrical position of the Spk in the hyphal apex of wild-type cells correlated with changes in the direction of growth elicited by surface topography. However, when the position of Spk travel over time was mapped onto final hyphal shape, there were numerous examples where its trajectory had diverged from the overall direction of growth (arrows in Fig. 7; Movie S4). These observations indicate that the position of the Spk is not an absolute predictor of the direction of growth in *C. albicans* and instead may lag behind the repositioning of other directional determinants.

## Discussion

### Contact-dependent tip asymmetry drives hyphal responses to surface topography

The production of hyphae by *C. albicans* is associated with tissue invasion in humans, but little is known about how its hyphal behaviours facilitate interactions with the host. Here we show that, during growth on surfaces as opposed to embedded conditions, *C. albicans* hyphal tips are morphologically asymmetrical and this extends to the intracellular positioning of key cell polarity complexes – the Spk, polarisome and exocyst – which translocate across the apex in response to touch. Our finding that the Spk orients towards the softer of two surfaces suggests that *C. albicans* may be able to sense surface stiffness. In *U. appendiculatus*, surface stiffness triggers spore germination (Terhune and Hoch, 1993) but it is not known how the mechanical properties of a surface are sensed by fungi.

**Figure 8:**
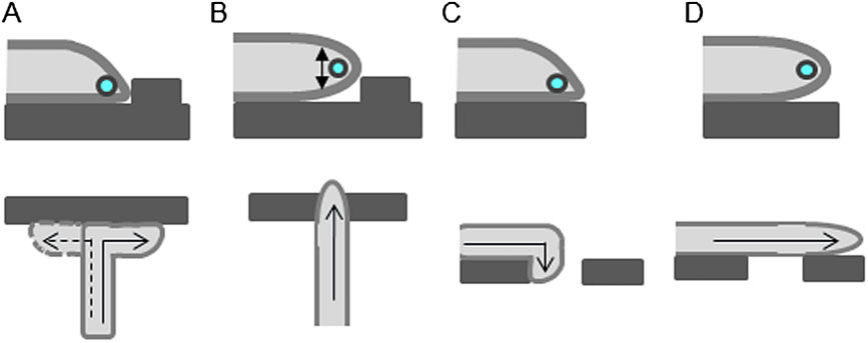
Model for the role of hyphal tip morphology in determining directional responses to surface topography.A. During growth on surfaces, hyphal tip asymmetry restricts growth to a site adjacent to the substratum. On encountering an obstacle, the reorientation of hyphal growth is permitted in the X–Y horizontal plane close to the surface. Wild-type hyphae are therefore forced to respond to relatively small obstacles by reorienting their direction of growth.B. The rounded tip morphology and meandering of the Spk in the vertical plane in *rsr1*Δ hyphae elevate the site of growth above the level of the substratum, thereby permitting growth over obstacles that would otherwise be perceived as barriers.C. An asymmetrical tip morphology drives growth towards the substrate so hyphal growth follows negative contours that slope away from the tip, permitting hyphal ingress into underlying depressions.D. In contrast, in cells that have not undergone tip contact or hyphae of the *rsr1*Δ mutant, where tip morphology is rounded, the penetration response into underlying discontinuities is lost.

Our results suggest a model whereby tip asymmetry is fundamental to the thigmotropic response to small obstacles because it positions the polarity machinery, and hence the growth site, close to the substratum. Encounters with obstacles can therefore only occur in left- or right-hand turns in the horizontal plane of the surface (Fig. 8A). Hyphae of the *rsr1*Δ mutant do not reorient thigmotropically (Brand *et al*., 2008). Our findings that *rsr1*Δ tips do not maintain an asymmetrical morphology and the Spk wanders within the tip suggests that the polarity machinery spends more time located above the substrate surface than in wild-type cells, thereby permitting *rsr1*Δ hyphae to grow straight over obstacles of less than half their diameter in height (Fig. 8B).

An equally important requirement for thigmotropism is frictional force between the hypha and the substrate. Tip contact alone was not sufficient to trigger reorientation. Instead, considerable pressure and mechanical stress had to be generated at the tip before reorientation occurred. This may contribute to pathogenicity, as discussed below, but the requirement for the forward thrust of tip growth to be countered by hyphal friction in order to generate tip pressure may explain why morphogenesis in *C. albicans* is accompanied by expression of multiple adhesins that are not present in yeast cells. The ability to adhere tightly to the host in order to generate penetrative force is an important factor in plant pathogenesis, where appressoria generate strong adhesion to the leaf surface so that extension of the penetration peg does not detach the fungus from its host (Bechinger *et al*., 1999).

Asymmetrical tip growth enabled *C. albicans* hyphae to follow contours ranging from gentle curves to acute bends of 90°, suggesting that this morphology plays a key role in probing underlying unconformities in the growth surface (Fig. 8C). Hyphae that had not undergone direct tip contact did not follow contours (Fig. 8D). Acute changes in growth direction were followed by a reversion to the initial overall growth trajectory when possible. This phenomenon has been termed ‘directional memory’ and has been observed in plant root hairs and *Neurospora crassa*, although not in pollen tubes (Bibikova *et al*., 1997; Held *et al*., 2011; Agudelo *et al*., 2013). This may be indicative of the nutrient foraging function of roots and hyphae, while pollen tubes are required to home into a specific target. Hyphae of the *rsr1*Δ mutant followed contours when the Spk was asymmetrically skewed towards the substrate, but when the Spk position in this mutant meandered its hyphae underwent random changes in growth trajectory. These two *rsr1*Δ populations are represented in Fig. 4F. The failure of the *rsr1*Δ mutant to remain closely apposed to the host surface and maintain a growth trajectory may contribute to its inability to invade host cell layers and hence its attenuated virulence (Yaar *et al*., 1997; Brand *et al*., 2008).

### The Spk is not an absolute predictor of growth direction

Previous studies of hyphal growth have suggested that the position of Spk is a predictor of the site of new tip growth. In this study, the Spk position was predictive of a specific growth response but we observed multiple examples of new growth occurring away from the site of the Spk. One possibility was that changes in hyphal growth direction were brought about by the incorporation of more cell wall material at the Spk side of the hyphal apex than the other. However, our observation that the Spk maintains a stable asymmetrical position during growth along a barrier without causing curvature of the hypha does not support this idea. The advent of molecular approaches coupled with improved live cell imaging may help to elucidate the relationship between Spk structure, function and directional growth responses.

### Hyphal tip force and adaptation to the host

The generation of penetrative force in human pathogenic fungi has not previously been studied. Many fungal plant pathogens produce specialist structures that penetrate plant cuticles by mechanical force alone. For example, the cereal pathogen, *Colletotrichum graminicola*, produces a penetration peg that exerts a force of 17 μN (Bechinger *et al*., 1999). The only filamentous human pathogen studied to date is the oomycete, *Pythium insidiosum*, which exerted a force of 2 μN and an applied pressure of 0.14 MPa (MacDonald *et al*., 2002). This was thought to be insufficient to penetrate human tissue without the aid of degradative enzymes because, using the little data available on human skin mechanics, it was calculated that the pressure required was an order of magnitude higher, i.e. 1–10 MPa (Bechinger *et al*., 1999; Ravishankar *et al*., 2001; Bastmeyer *et al*., 2002). The measurement of force exerted by the hyphae of small fungi such as *C. albicans* is technically challenging. Indentation of silicone elastomer was used to establish a growth stalling force for *Schizosaccharomyces pombe* of 11.3 μN, using the published Young's modulus of the elastomer (Minc *et al*., 2009). Here, we used atomic force microscopy to generate a standard curve of force versus indentation distance specific to the silicone elastomer we used. We estimated the force applied by the *C. albicans* tip to be ∼ 8–9 μN, with a resulting tip pressure of ∼ 1.2 MPa. The force required to pierce a murine oocyte with an IVF needle of 5 μm diameter was reported as 7.5 μN, or 13.0 μN for an embryo (Sun *et al*., 2003). Our measurements fell within this range so it seems likely that *C. albicans* hyphae can penetrate host cell membranes using mechanical force alone. Consistent with this, *C. albicans* hyphae growing in biofilms are known to penetrate soft medical silicone, which has a Young's modulus similar to that of human cartilage (Leonhard *et al*., 2013). The mechanical piercing of the murine oocyte with a needle distended the plasma membrane by 45 μm before it was finally breached (Sun *et al*., 2003). A similar distension of the macrophage membrane prior to its breaching by *C. albicans* hyphae has been observed (McKenzie *et al*., 2010), suggesting that mechanical force alone is operating in this context. However, enzyme activity may be important for efficient penetration of certain tissues *in vivo*, where inward distention of epithelial cells by hyphae has been observed but its depth has not been measured (Dalle *et al*., 2010).

The observation that hyphal reorientation occurs at tip pressures higher than that required for plasma membrane penetration suggests that *C. albicans* hyphae are programmed to attempt tissue penetration but reorient if greater resistance is met. If hyphal reorientation was triggered at a lower tip pressure, hyphae would not be able to penetrate host cell membranes. Other aspects of *C. albicans* hyphal biology may specifically support its penetrative ability. For example, the bread mould fungus, *N. crassa*, exerts a maximum force and pressure of only 3.2 μN and 0.25 MPa, respectively, and undergoes tip splitting on perpendicular contact with an obstacle (Money *et al*., 2004; Held *et al*., 2011). The lack of tip-splitting behaviour in *C. albicans* may be a further biological feature that helps the pathogen to apply the pressure required to infiltrate the tissue of its host.

Together, these findings suggest how the emergent properties of polarized hyphal growth result in exploratory behaviours that are likely to be fundamental to all filamentous fungal pathogens.

## Experimental procedures

### Strains and growth media

The *C. albicans* strains and standard culture methods used in this study are detailed in Supplementary Table S1 and Supplementary Methods.

### Preparation of PDMS and glass substrates

PDMS (Sylgard-184, Dow Corning) was mixed at a curing agent to elastomer ratio of 1:10, de-gassed, cast in a 10 cm^2^ Petri dish (no patterning, for adhesion assays) or on a nickel shim with nano-fabricated obstacles of 1.05 or 1.5 μm (Kelvin Nanotechnology, Glasgow, UK) and cured for 2 h at 60°C. Chemical modification of PDMS surfaces: UVO_3_ treatment for 30 min (Jelight) or gold sputtering at 25 mA for 1 min with a Sputter Coater K550 (EMITECH) with overnight coating with Collagen I (0.05 mg ml^−1^ in phosphate-buffered saline (PBS) pH 7.0), Collagen IV (0.05 mg ml^−1^ in PBS pH 7.0), poly-L-lysine (0.01% in ddH_2_O) or fibronectin (0.05 mg ml^−1^ in ddH_2_O) as indicated. Substrates were dried for 24 h at RT. Surface modification was validated by quantifying changes in contact angle using the sessile drop method on a goniometer (First Ten Angstroms FTA1000, V2.1), and by X-ray photoelectron spectroscopy of key samples (see Supplementary Methods). Glass substrates for asymmetry experiments were silanized by acid washing in 1.0 M HCl and treating with 5% dimethylsilane in xylene for 5 min before drying, further washing and sterilizing at 200°C. The mean contact angles [± standard deviation (SD)] of untreated or silanized glass were 68 ± 6.4° and 91 ± 4.5° respectively.

### Adhesion and thigmotropism assays

For adhesion assays, yeast cell concentrations were normalized and cells adhered to PDMS surfaces in dH_2_O for 30 min at room temperature before washing to remove non-adhered cells. Results were reported as cells adhered per mm^2^. For thigmotropism assays, adhered cells were incubated in 20% FCS, 2% glucose at 37°C for 6 h in Petri dishes. Results were reported as the mean percentage (± SD, *n* = 6) of hypha-ridge interactions resulting in tip reorientation. Images were captured with an Olympus BX50 (Olympus) fitted with a Lumenera Infinity 1 CMOS (Lumenera) camera.

### Determination of force and pressure values

Indentation distance of hyphal tips into PDMS was measured by DIC microscopy from time lapse images to ascertain the point of maximal indentation. Measurements were used to extrapolate force values from a standard curve of force versus indentation distance, generated by atomic force microscopy (see Fig. S2 for methods). Hyphal tip pressure was derived from the equation *P* = *F*/*A*, where *F* is the force and *A* is the hyphal tip surface contact area, which was assumed to be hemispherical. Contact area (*A*) was calculated as 2π*Rr*, where *R* was the 1.2 μm radius of a hypha and *r* was the indentation distance.

### Live cell imaging

Yeast cells in 20% FCS and 2% glucose were spread on 1.5 cm^2^ PDMS adhered to a microscope slide, and sealed under a coverslip using Velapa (1:1:1 Vaseline: lanolin: paraffin wax; Veses and Gow, 2008). The slide was incubated at 37°C on an inverted Zeiss AxioObserver Z1 running Axiovision (v. 4.8; Carl Zeiss) with incubator hood (PeCon GmbH). Multi-point time lapse images were acquired using a Plan-Neofluar 40×/1.3NA oil immersion objective lens (Carl Zeiss) and a 16 bit CoolSNAP H^2^ CCD camera (Photometrics). Z-stacks were acquired at Nyquist sampling rates and deconvolved using Axiovision. GFP was imaged using a Zeiss #62 high efficiency (HE) B/G/HR filter cube. YFP was imaged using a Zeiss #60 HE C/Y/HR filter cube or a Laser 2000 YFP filter (Semrock). Image analysis was performed using ImageJ (v. 1.45s; NIH) or Volocity (v. 6.1; Perkin Elmer) for three-dimensional rendering. See Supplementary Methods for image analysis methods.

### Statistical analyses

Data were analysed using Prism 5.04 (GraphPad). Two-tailed independent Student's *t*-tests were used for tip reorientation time (*n* = 74) and indentation distance (*n* = 61). Thigmotropism experiments were analysed by analysis of variance with a post-hoc Dunnett's *t*-test (*n* = 6 independent experiments). Correlation analysis was used for directional memory assays (*n* = 67). A chi-square test was used for analysing asymmetry on surfaces (*n* = 18).

## Supporting Information

**Fig. S1.** Topographical features cast in Sylgard-184 PDMS elastomer. Topographical features are 1 μm high. Measurements of obstacles are shown in micrometre. The smallest channels were 2 μm wide and the offset between gaps in ridges was 10 μm. **Fig. S2.** Standard curve of force versus indentation distance in PDMS elastomer. The standard curve used to extrapolate hyphal force values was generated by Atomic Force Microscopy using a Bioscope DAFMLN head (Bruker) mounted on an Axiovert 100 microscope (Zeiss). A cantilever with a spring constant of 14 N m^−1^ fitted with a borosilicate glass bead of 2 μm diameter (Novascan) was used to indent five areas for each of 15 samples. The indentation distance for wild-type cells of 950 ± 40 nm falls at the top of the slope (broken line), so the value calculated for the force (8.7 nN) is likely to be the minimum applied by a hypha. For the *rsr1*Δ mutant, the indentation distance was 876 ± 40 nm (dashed broken line). **Fig. S3.** Chemical analysis of untreated and chemically modified PDMS surfaces. A. The sessile drop method was used to measure the contact angle of water on PDMS samples and *C. albicans* yeast cells. Untreated PDMS was hydrophobic. *C. albicans* wild-type cells (hatched bar) were hydrophilic. Bars = SD. B. The surface chemical composition as the atomic percentage of the major chemical components of the PDMS (O, Si, C), gold (AU) and protein (N) was examined on the key PDMS surface chemistries using XPS. PDMS exposed to UVO_3_ (UV ozone) showed an increase in oxygen content, consistent with the formation of SiO_x_ groups on the surface. Au was present on the surface of gold-sputtered PDMS and was detectable in smaller amounts after coating with Collagen IV. The higher percentage of Collagen IV adsorbed to gold compared with UVO_3_-treated PDMS could underlie the increased adhesion of *C. albicans* cells to this surface. n.d., not detected. **Table S1.** *Candida albicans* strains used in this study. **Table S2.** Primers used in this study.

Movie S1 Time-lapse movie of immobilised hypha re-orienting on contact with a barrier in an enclosed imaging chamber 1 µm in height.

Movie S2 The Spk translocates across the hyphal apex in response to new tip contact. Hyphae were grown in a live-cell imaging chambers and imaged using DIC and fluorescence microscopy.

Movie S3 Hyphae were grown in live-cell imaging chambers featuring barriers with gaps. Hyphae resumed the original growth trajectory (arrows) after temporary deflection by an obstacle.

Movie S4 Time-lapse images of Spk oscillation in hyphal tips that contacted a barrier perpendicularly. The Spk is indicated by expression of Mlc1-GFP.

Movie S5 Time-lapse movie of a non-immobilised hypha contacting a barrier in an enclosed chamber 1.5 µm in height.
