# Supplementary material for: Contact-induced apical asymmetry drives the thigmotropic responses of Candida albicans hyphae
Source: Cell Microbiol. 2014 Nov 25;17(3):342–54. doi: 10.1111/cmi.12369 (PMC4371639; doi:10.1111/cmi.12369)
Supplement: Supplementary file 1 — Fig. S1. Topographical features cast in Sylgard-184 PDMS elastomer. Topographical features are 1 μm high. Measurements of obstacles are shown in micrometre. The smallest channels were 2 μm wide and the offset between gaps in ridges was 10 μm. Fig. S2. Standard curve of force versus indentation distance in PDMS elastomer. The standard curve used to extrapolate hyphal force values was generated by Atomic Force Microscopy using a Bioscope DAFMLN head (Bruker) mounted on an Axiovert 100 microscope (Zeiss). A cantilever with a spring constant of 14 N m−1 fitted with a borosilicate glass bead of 2 μm diameter (Novascan) was used to indent five areas for each of 15 samples. The indentation distance for wild-type cells of 950 ± 40 nm falls at the top of the slope (broken line), so the value calculated for the force (8.7 nN) is likely to be the minimum applied by a hypha. For the rsr1Δ mutant, the indentation distance was 876 ± 40 nm (dashed broken line). Fig. S3. Chemical analysis of untreated and chemically modified PDMS surfaces. A. The sessile drop method was used to measure the contact angle of water on PDMS samples and C. albicans yeast cells. Untreated PDMS was hydrophobic. C. albicans wild-type cells (hatched bar) were hydrophilic. Bars = SD. B. The surface chemical composition as the atomic percentage of the major chemical components of the PDMS (O, Si, C), gold (AU) and protein (N) was examined on the key PDMS surface chemistries using XPS. PDMS exposed to UVO3 (UV ozone) showed an increase in oxygen content, consistent with the formation of SiOx groups on the surface. Au was present on the surface of gold-sputtered PDMS and was detectable in smaller amounts after coating with Collagen IV. The higher percentage of Collagen IV adsorbed to gold compared with UVO3-treated PDMS could underlie the increased adhesion of C. albicans cells to this surface. n.d., not detected. Table S1. Candida albicans strains used in this study. Table S2. Primers used in this study. [file cmi0017-0342-sd1.docx]

**Cellular Microbiology**

**Supplemental Information**

**Contact-induced apical asymmetry drives the thigmotropic responses of *Candida albicans* hyphae**

Darren D. Thomson, Silvia Wehmeier, Fitzroy J. Byfield, Paul A. Janmey, David Caballero-Lima, Alison Crossley and Alexandra C. Brand*

*School of Medical Sciences, University of Aberdeen, Foresterhill, Aberdeen AB25 2ZD

Email: [a.brand@abdn.ac.uk](mailto:a.brand@abdn.ac.uk) Tel: +44-1224-437495

**Supplemental Experimental Procedures**

### *Strains and growth conditions*

Strains used in this study are shown in Supplementary Table S1. Yeast cells were grown overnight at 30°C with shaking at 200 rpm in YPD (1 % (w/v) yeast extract, 2 % (w/v) mycological peptone, 2 % (w/v) glucose), with 2 % (w/v) agar for solid medium. For live-cell imaging in enclosed chambers and for open-dish thigmotropism assays, cells were pelleted, washed with ddH_2_O, inoculated at a 1:100 dilution onto the appropriate substratum and incubated in 2 % glucose, 20 % FCS to induce hyphal growth.

*Strain construction*

Kel1-YFP was derived from BWP17 (ura3Δ-*iro1*Δ::λimm434/ura3Δ-*iro1*Δ::λimm434, *his1*::*hisG/his1*::*hisG arg4*::*hisG/arg4*::*hisG*) (Wilson *et al*., 2000). YFP was C-terminally fused to both copies of Kel1 by amplifying YFP from plasmids pFA-YFP-HIS1 and pFA-YFP-ARG4 (Gola *et al.,* 2003) with primers S1 KEL1 XFP and S2 KEL1 XFP, where homology with the plasmid is indicated in lower case (Supplementary Table S2). Transformations were carried out using the lithium acetate method (Walther and Wendland, 2003). Strains were confirmed by PCR using forward primers H3FA and A3FA with reverse primer G4-KEL1. Expression of the fusion protein was verified by western blot using α-GFP Living colors A.v. Monoclonal Antibody (JL-8) (Clontech, USA). Oligonucleotides were obtained from biomers.net (Ulm, Germany).

*X-ray Photoelectron Spectroscopy (XPS)*

PDMS surface mofidication was verified using XPS analysis. XPS was performed in an ion pumped VG Microtech CLAM 4 MCD analyser system using 200 Watt unmonochromated Mg X-ray excitation. Samples supported on carbon pads on stubs were introduced into the instrument via a turbo molecular pumped entry lock, which was pumped for 15 minutes before the sample was introduced into the analysis chamber. The slit (5 mm) was used with no apertures selected. The analyser was operated at constant pass energy of 100 eV for wide scans and 20eV for detailed scans setting the C1s peak at BE 284.8 eV to overcome any sample charging. Data was obtained using SPECTRA Version 8 operating system. Data processing was performed using CASAXPS. Peak areas were measured after satellite subtraction and background subtraction either a linear background or following methods of Shirley, 1972 (Shirley, 1972).

*Image Analysis*

**Substrate-preference analysis**: deconvolved Z-stacks were imported into Volocity (v. 6.1; Perkin Elmer, Massachusetts, USA) for 3D-rendering in DIC and fluorescence channels. FP-tagged protein localization was assigned as situated within the top, middle or bottom third of the rendered tip with respect to the substrates used - ridged PDMS, flat PDMS or glass-bottom µ-dishes (Ibidi, Matinsried, Germany). **Hyphal tip indentation distance** into PDMS was determined using ImageJ software. **Changes in hyphal growth trajectory** on encountering obstacles were quantified using AxioVision LE (Zeiss) to record angle of approach and angle of exit relative to a line perpendicular to the object (Fig. 4B). **Fluorescence maxima plots** were generated from time lapse movies using ImageJ. The background signal in each frame of the fluorescent channel was subtracted using a rolling ball radius of 50 pixels, followed by the application of a 1 sigma radius Gaussian blur. The fluorescence maxima for each frame was determined and maximally projected onto the final DIC frame of the time lapse movie.

**Table S1: *C. albicans* strains used in this study**

| **Strain** | **Published name** | **Genotype** | **Source** |
| --- | --- | --- | --- |
| CAI4/CIp10 | NGY152 | *ura3*Δ*-iro1*Δ*::imm434/*Δ*ura3*Δ*-iro1*Δ*::imm434 RPS1/RPS1-Clp10-URA3* | Murad *et al.,* 2000 |
| BWP17 | BWP17 | ura3Δ-*iro1*Δ::λimm434/ura3Δ-*iro1*Δ:: λimm434, *his1*::*hisG/his1*::*hisG arg4*::*hisG/arg4*::*hisG* | Wilson *et al.*, 2000 |
| Mlc1-YFP | YMG7139 | As BWP17:  *MLC1/MLC1-YFP::URA3* | Crampin *et al*., 2005 |
| Spa2-YFP | YMG6748 | As BWP17: *SPA2/SPA2-YFP-URA3* | Crampin *et al*., 2005 |
| Exo70-YFP | Exo70-YFP | As BWP17:  *EXO70/EXO70-YFP-URA3* | Jones and Sudbery,  2010 |
| Kel1-YFP | DCL201 | As BWP17:  *KEL1-YFP-HIS1/KEL1-YFP-ARG4* | This study |
| Bni1-GFP | WYL5 | As BWP17:  *bni1Δ ::ARG4/BNI1-GFP ::URA3* | Li *et al.,* 2005 |
| LifeAct  *rsr1*Δ | DCL790  CA9151 | As BWP17:*TEF1*/*Δ* LIFEACT-GFP::*HIS1*  As BWP17: rsr1::ARG4/rsr1::HIS1 MLC1/MLC1-YFP-  URA3 | Sudbery, 2011  Pulver et al., 2013 |
|  |  |  |  |

**Table S2: Primers used in this study**

| **Name** | **Sequence 5’🡪3’** |
| --- | --- |
| S1 KEL1 XFP | AGACTTGGAAGCTGACTTGTATATATTGAAACAAGAAAGAGATCAATTAAAAGACAATGTCACTTCGTTGCAAAAACAACTTTATTTAGCTCAGAATCAAggtgctggcgcaggtgcttc |
| S2 KEL1 XFP | GAACAACCAAGTCCAGTCAACATGACTTCGAATCAACCATTCTCGTTTTTTACATACCAGAACACCAACACGTCCCCGCCAGTCCCATTGGATGACGtctgatatcatcgatgaattcgag |
| G4-KEL1 | GCCGCTGTATGTGCCACCAG |
| H3FA | GGACGAATTGAAGAAAGCTGGTGCAACCG |
| A3FA | GGGCCCATTGGTTAAGTTCATATGC |
|  |  |
|  |  |

**Supplemental References**

Crampin, H., Finley, K., Gerami-Nejad, M., Court, H., Gale, C., Berman, J., and Sudbery, P. (2005) *Candida albicans* hyphae have a Spitzenkörper that is distinct from the polarisome found in yeast and pseudohyphae**.** *J Cell Sci* **118***:* 2935-2947.

Gola, S., Martin, R., Walther, A., Dϋnkler, A. and Wendland, J. (2003) New modules for PCR-based gene targeting in *Candida albicans*: rapid and efficient gene targeting using 100 bp of flanking homology region**.** *Yeast* **20***:* 1339-1347.

Jones, L.A. and Sudbery, P.E. (2010) Spitzenkörper, exocyst, and polarisome components in *Candida albicans* hyphae show different patterns of localization and have distinct dynamic properties**.** *Eukaryot Cell* **9***:* 1455-1465.

Li, C.R., Wang, Y.M., De Zheng, X., Liang, H.Y., Tang, J.C.W., and Wang, Y. (2005) The formin family protein CaBni1p has a role in cell polarity control during both yeast and hyphal growth in *Candida albicans***.** *J Cell Sci* **118**: 2637-2648.

Murad, A.M., Lee P.R., Broadbent I.D., Barelle, C.J., and Brown, A.J. (2000). CIp10, an efficient and convenient integrating vector for *Candida albicans*. *Yeast* **16***:* 325-327

Pulver, R., Heisel, T., Gonia, S., Robins, R., Norton, J., Haynes, P., and Gale, C.A. (2005). Rsr1 focuses Cdc42 activity at hyphal tips and promotes maintenance of hyphal development in *Candida albicans*. *Eukaryot Cell* **4**: 1273-1281.

Shirley, D.A. (1972) High-resolution X-ray photoemission spectrum of the valence bands of gold**.** *Phys Rev B* **5***:* 4709-4714.

Sudbery P.E. (2011) Growth of C*andida albicans* hyphae**.** *Nat Rev Micro* **9**: 737-748.

Walther, A., and Wendland, J. (2003) An improved transformation protocol for the human fungal pathogen *Candida albicans***.** *Curr Genet* **42**: 339-343.

Wilson, R.B., Davis, D., Enloe, B.M., and Mitchell, A.P. (2000) A recyclable *Candida albicans URA3* cassette for PCR product-directed gene disruptions. *Yeast* **16**: 65-70.

**
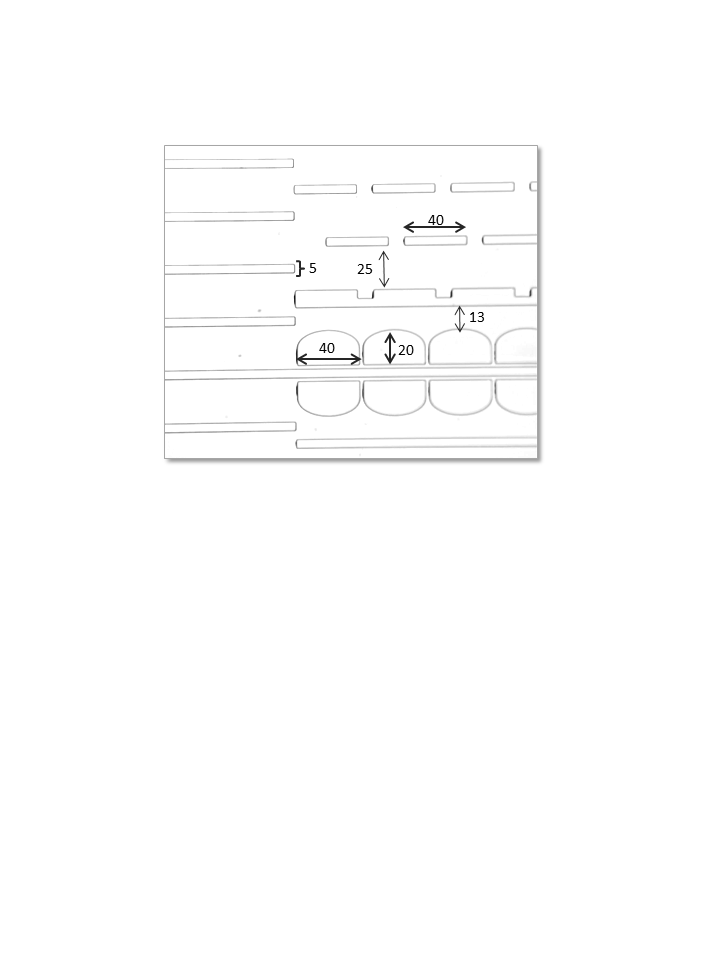
**

**Fig. S1.** **Topographical features cast in Sylgard-184 PDMS elastomer.** Topographical features are 1 µm high. Measurements of obstacles are shown in µm. The smallest channels were 2 µm wide and the offset between gaps in ridges was 10 µm.

**Fig. S2. Standard curve of force vs indentation distance in PDMS elastomer.** The standard curve used to extrapolate hyphal force values was generated by Atomic Force Microscopy using a Bioscope DAFMLN head (Bruker, Billerica, MA) mounted on an Axiovert 100 microscope (Zeiss, Thornwood, NY).  A cantilever with a spring constant of 14 N/m fitted with a borosilicate glass bead of 2 μm diameter (Novascan, Ames, IA) was used to indent 5 areas for each of 15 samples. The indentation distance for wild-type cells of 950 ± 40 nm falls at the top of the slope (broken line), so the value calculated for the force (8.7 nN) is likely to be the minimum applied by a hypha. For the *rsr1*Δ mutant, the indentation distance was 876 ± 40 nm (dashed broken line).

**
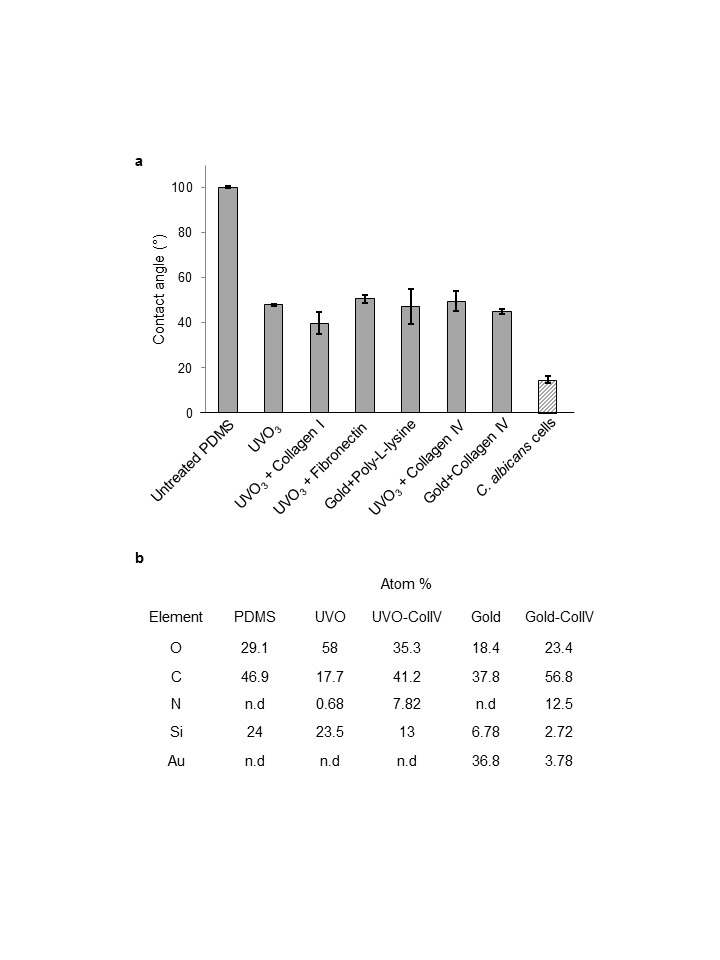
**

**Figure S3:** **Chemical analysis of untreated and chemically-modified PDMS surfaces**. (**a**) The sessile drop method was used to measure the contact angle of water on PDMS samples and *C. albicans* yeast cells. Untreated PDMS was hydrophobic*.*   *C. albicans* wild-type cells (hatched bar) were hydrophilic. Bars = SD. (**b**) The surface chemical composition as the atomic percentage of the major chemical components of the PDMS (O, Si, C), gold (AU) and protein (N) was examined on the key PDMS surface chemistries using XPS. PDMS exposed to UVO_3_ (UV-ozone) showed an increase in oxygen content, consistent with the formation of SiO_x_ groups on the surface. Au was present on the surface of gold-sputtered PDMS and was detectable in smaller amounts after coating with Collagen IV. The higher percentage of Collagen IV adsorbed to gold compared to UVO_3_ –treated PDMS could underlie the increased adhesion of *C. albicans* cells to this surface. n.d. = not detected.
